# FALLS PREDICT FASTER PROGRESSION OF ALZHEIMER DEMENTIA

**DOI:** 10.1093/geroni/igae098.3977

**Published:** 2024-12-31

**Authors:** Audrey Keleman, Melody Li, Rebecca Bollinger, Melissa Krauss, Elizabeth Grant, John Morris, Susan Stark

**Affiliations:** Department of Veterans Affairs, St. Louis, Missouri, United States; Washington University in St. Louis, St. Louis, Missouri, United States; Washington University in St. Louis, St. Louis, Missouri, United States; Washington University in St. Louis, St. Louis, Missouri, United States; Washington University in St. Louis, St. Louis, Missouri, United States; Washington University in St. Louis, St. Louis, Missouri, United States; Washington University in St. Louis, St. Louis, Missouri, United States

## Abstract

Preclinical Alzheimer disease (AD) is a decades-long period in which pathological changes accumulate in the brain (e.g., amyloid plaques) but individuals remain cognitively unimpaired (CU; Clinical Dementia Rating®[CDR®]=0). Motor impairments such as falls have been associated with preclinical AD and may offer early intervention opportunities. However, as aging affects cognition, motor function, and falls, differentiating between trajectories of healthy aging and preclinical AD is complex. This longitudinal cohort study investigated the impact of falls and preclinical AD on dementia symptom onset. We prospectively monitored falls for 1 year among 124 CU older adults and assessed preclinical AD status using amyloid positron emission tomography. We followed this cohort for 12 years and evaluated CDR annually. The cohort was grouped by baseline preclinical AD and faller status: 34 Preclinical AD-/Fall- (“healthy agers”), 53 Preclinical AD-/Fall+, 15 Preclinical AD+/Fall-, and 22 Preclinical AD+/Fall+. Kaplan-Meier survival analysis examined time to progression to CDR 1 (mild dementia) by group. Participants were on average 74 years old at baseline, 78% female, and 96% White with no significant demographic differences between groups. Preclinical AD+/Fall+ individuals progressed to CDR 1 most rapidly, while Preclinical AD-/Fall- (“healthy agers”) progressed least quickly. Preclinical AD+/Fall- and Preclinical AD-/Fall+ groups had similar progression rates. Each group’s proportional hazard of dementia progression differed significantly from healthy agers, unadjusted and controlling for age and gender (ps< 0.5). These findings suggest falls associate with faster progression of AD dementia, warranting further research to disentangle motor function and falls in healthy agers and those with preclinical AD.

